# Late Bayesian inference in mental transformations

**DOI:** 10.1038/s41467-018-06726-9

**Published:** 2018-10-24

**Authors:** Evan D. Remington, Tiffany V. Parks, Mehrdad Jazayeri

**Affiliations:** Department of Brain & Cognitive Sciences, McGovern Institute for Brain Research, Massachusetts Institute of Technology, Cambridge, MA 02139 USA

## Abstract

Many skills rely on performing noisy mental computations on noisy sensory measurements. Bayesian models suggest that humans compensate for measurement noise and reduce behavioral variability by biasing perception toward prior expectations. Whether a similar strategy is employed to compensate for noise in downstream mental and sensorimotor computations is not known. We tested humans in a battery of tasks and found that tasks which involved more complex mental transformations resulted in increased bias, suggesting that humans are able to mitigate the effect of noise in both sensorimotor and mental transformations. These results indicate that humans delay inference in order to account for both measurement noise and noise in downstream computations.

## Abstract (web summary)

Humans compensate for sensory noise by biasing sensory estimates toward prior expectations, as predicted by models of Bayesian inference. Here, the authors show that humans perform ‘late inference’ downstream of sensory processing to mitigate the effects of noisy internal mental computations.

## Introduction

**Fig. 1:**
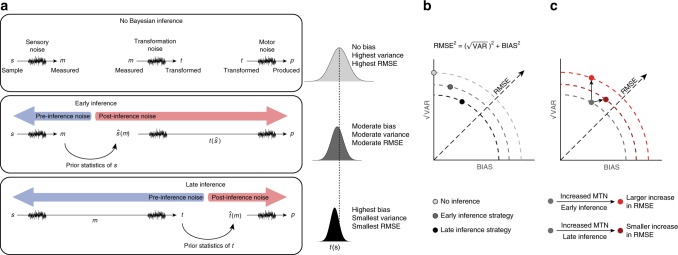
Inference in the presence of sensory and mental transformation noise (MTN). **a** Observer models. Top: no inference. The observer makes a noisy measurement (*m*) of a sensory stimulus (*s*), applies a noisy mental transformation to compute *t*, and aims to produce *t* through a noisy motor system resulting in *p*. Across trials, *p* has a distribution (light gray) centered on the correct value (dashed line). Middle: early inference. After the noisy measurement, the observer uses the prior statistics of *s* to infer $\hat s$, uses $\hat s$ to generate *t*, and uses *t* to produce *p*. Since the inference is made before the transformation (early inference), prior-dependent biases only reduce the effect of sensory noise that precedes the inference (pre-inference noise, blue arrow), and not the uncertainty due to the post-inference transformation noise (red arrow), which directly increases variability. In this case, the distribution of *p* (intermediate gray) is biased but has lower variance. Bottom: Late inference. The inference is made after the transformation based on the prior statistics of *t* to generate an optimal inference, $\hat t$, which is then used to produce *p*. Late inference leads to larger biases but allows the observer to account for both sensory and transformation noise. The distribution of *p* values is more biased and less variable than the early inference strategy (dark gray bell-shaped distribution). **b** Variability (√VAR), bias (BIAS), and RMSE for the three observer models in panel **a**. The sum of squares relationship between √VAR, RMSE, and BIAS (top) can be depicted on a quarter circle (dashed lines) with the radius representing RMSE. Compared to the no inference strategy (light gray), early inference (medium gray) increases BIAS while reducing √VAR and RMSE. Late inference (dark gray) further increases BIAS and minimizes RMSE. **c** Effects of MTN on behavior. In early and late inference, additional MTN primarily increases √VAR and BIAS, respectively (compare bright and dark red to gray). Crucially, the late inference leads to an overall smaller increase in RMSE (smaller radius of the dark circle compared to the bright one). See the Supplementary Figures 1-3 and Supplementary Methods for a more detailed discussion of the effects of pre- and post-inference noise on BIAS and √VAR and a quantitative comparison of the early and late inference models

When putting sensory information to use in the control of behavior, the brain has to measure sensory information, convert that information to behaviorally relevant variables, and use those variables to guide actions. For example, to catch a ball, the brain has to measure the position and velocity of the ball, convert those measurements to an appropriate movement plan, and then move the hand to catch the ball. Noise in sensory measurements, noise during the transformation of sensory measurements to behaviorally relevant variables, and noise in the motor system can all contribute to outcome uncertainty (Fig. 1a, top). Research in the past several decades has demonstrated that the brain incorporates knowledge about sensory and motor noise to optimize behavior. For example, when multiple sensory cues are available, humans rely more heavily on cues that are more reliable^1–4^. Similarly, humans use their prior knowledge of the statistics of sensory inputs to improve sensory estimates^5–8^. It has also been shown that humans optimize movement trajectories by taking into account motor noise and expected outcomes^9,10^. These and related observations in multiple modalities^11–15^ have provided strong evidence that the brain implements strategies to reduce the effect of noise in sensory and motor systems on behavior.

Far less is known about whether and how humans account for noise associated with the intervening stage of transforming sensory measurements to behaviorally relevant variables. The ability to apply such transformations reliably is central to our behavioral repertoire and to the performance of athletes, musicians, and professionals such as surgeons and airplane pilots. For example, consider tracing an image while looking at the reflection of your hand in a mirror. This is a challenging task, not because it is hard to see or to draw, but because of the need to apply a mental transformation to handle the inversion caused by the mirror. Another example is the challenge of deciding whether two objects viewed from different angles are the same^16^. In this case, in addition to processing the visual features, one must mentally “rotate” one or both objects to decide their similarity. Such sensorimotor and mental transformations are ubiquitous and can powerfully impact behavior^13,17–22^.

Motivated by the success of normative models showing that humans use prior expectations to optimize behavior in the presence of sensory noise, we hypothesized that humans might be able to minimize the effects of additional noise in sensorimotor and mental transformations (hereafter, mental transformation noise, MTN) by relying on the statistical regularities associated with the outcome of those transformations. Importantly, this hypothesis bears on where in the brain inferences are made. On one hand, the brain might employ an “early inference” strategy in which estimates are computed before a mental transformation is applied (Fig. 1a, middle). This would bias estimates toward prior expectations of the stimulus and reduce uncertainty associated with the pre-inference sensory noise in the system^5,23,24^. In some cases, it may be possible to introduce additional bias to an early inference strategy to moderately reduce the effect of certain kinds of post-inference noise^25,26^. However, biases prescribed by an early inference strategy cannot mitigate variability caused by post-inference MTN (Supplementary Figures 1–2). On the other hand, the brain might adopt a “late inference” strategy and generate inferences after the transformation stage as a part of motor planning and decision making (Fig. 1a, bottom). Delaying inference in this way would improve performance relative to the early inference strategy by generating biases that can suitably mitigate the effects of both sensory noise and MTN (Fig. 1b, Supplementary Figure 3 and Supplementary Methods).

The early and late inference strategies make distinct predictions about the effect of MTN on behavior (Fig. 1c). With an early inference strategy, larger MTN would increase post-inference noise and lead to comparable increases in behavioral variability. In contrast, a late inference strategy in which MTN contributes to pre-inference noise would avoid much of this variance and improve performance by introducing additional biases toward the mean of the prior distribution (Supplementary Figures 1–3 and Supplementary Methods). Importantly, additional bias toward prior expectations due to larger MTN is inconsistent with an early inference strategy, and would constitute evidence that the brain makes inferences after sensorimotor and mental transformations (Fig. 1c).

Previous studies that only varied sensory and/or motor noise were unable to distinguish between early and late inference strategies. To distinguish between these two possibilities, it is critical to vary MTN independently from changes in the sensory and motor noise. To systematically vary MTN, we exploited the observation that more complex mental transformations engender more noise^13,17,18,20,21^. We devised several tasks, three sensorimotor and one perceptual, to test our hypothesis. In each task, we compared performance between two contexts. In one context, which we refer to as the “identity context”, the target quantity matched a previously measured quantity. This was compared to a more complex “remapped context” in which correct responses required subjects to apply a non-identity transformation to the sensory quantity. For example, subjects had to produce a duration that was 50% longer than the stimulus. As expected, the remapped context negatively impacted performance in all tasks, revealing the degrading effect of MTN. However, increases in MTN in the remapped context did not simply increase behavioral variability; instead, it led to increased biases toward the mean of the prior, an indication of the late Bayesian inference strategy that takes MTN into account.

## Results

### Summary of task structure

We conducted four psychophysical experiments, three involving sensorimotor tasks, and one involving a perceptual task. For the sensorimotor tasks, trials consisted of two epochs: a measurement epoch during which a sensory quantity (time interval or angle) was measured, and a subsequent production epoch during which subjects had to produce a quantity based on the preceding measurement. For each task, 11 or 12 subjects were tested in two contexts: an identity context in which the produced quantity had to match the sensory quantity, and a remapped context in which subjects had to apply a linear transformation to the sensory quantity to compute the desired produced quantity. For the perceptual task, the structure was similar to the sensorimotor tasks. Subjects first measured a sensory quantity (length) then provided a multiple choice response.

### Experiment 1: Ready, Set, Go with gain of 1 and 1.5

**Fig. 2:**
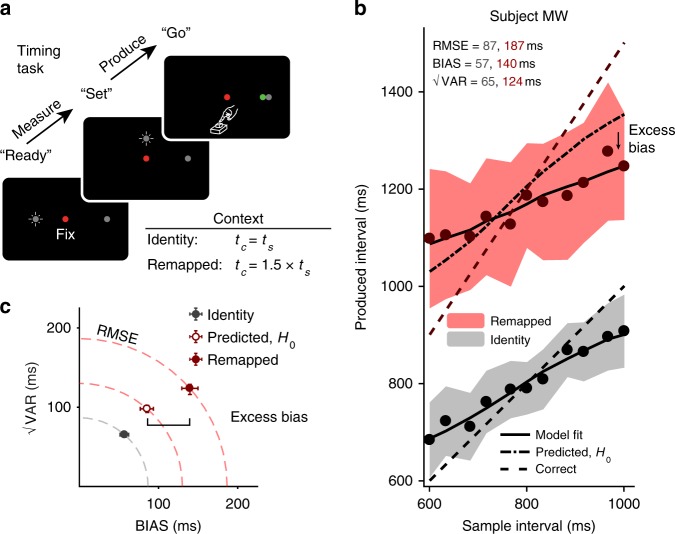
Time measurement and production task, gain = 1 and 1.5. **a** Trial structure. Each trial began with the presentation of a red fixation spot and a gray “Go target”. Subjects had to measure a sample time interval *t*_*s*_ demarcated by two flashes (“Ready” and “Set”). After Set, subjects had to press a key (“Go”) to produce an interval as close as possible to the correct interval *t*_*c*_ = gain × *t*_*s*_. In the “identity” context, the correct interval was the same as *t*_*s*_ (gain = 1), whereas in the “remapped” context, the gain was 1.5. The ratio between the distances of Go and Ready to the fixation point was equal to the gain factor. After the response, subjects received feedback via a colored circle, whose position relative to the Go target indicated the magnitude and sign of the error. The color of the circle indicated whether the error was smaller than a fixed threshold (green for a “hit,” white for a “miss”; the example illustrates a hit trial). **b** Behavior. Produced interval of a representative subject in the identity (gray) and remapped (red) contexts (filled circles: mean, shaded regions: mean ± one standard deviation; dashed line: correct intervals). Solid lines represent the mean responses of a Bayesian observer-actor model (see Methods) fit to the subject’s data separately for the two contexts; the dash-dot line in the gain = 1.5 condition corresponds to the prediction for the remapped context under the null hypothesis, using parameters of the model fit to the identity context (*H*_0_: no additional MTN). The subject’s behavior shows excess bias beyond what was predicted assuming no additional MTN. **c** √VAR vs. BIAS for the two contexts (gray: identity, dark red: remapped), as well as the prediction for the remapped context assuming no additional MTN (empty circle). This prediction underestimates RMSE indicating larger MTN for the remapped context. A substantial portion of increased RMSE was due to an increase in BIAS (“excess bias”). Dashed quarter circles illustrate combinations of BIAS vs. √VAR giving rise to equal RMSE; error bars represent 95% confidence intervals estimated using a bootstrap procedure (*n* = 1000)

Human subjects performed a time interval measurement and production task (Fig. 2a), also known as the “Ready, Set, Go” task, similar to a previous study^8^. During the measurement epoch, subjects were presented with a sample interval (*t*_*s*_; see Table 1 for all variables and abbreviations) demarcated by two visual flashes, “Ready” and “Set” (Fig. 2a). Subjects had to measure *t*_*s*_ and produce an interval (*t*_*p*_) afterwards by a key press (“Go”, no flashed stimuli). The interval *t*_*p*_ was measured from the start of the Set flash until the key press. In both identity and remapped contexts, *t*_*s*_ was drawn from the same discrete uniform prior distribution with 11 values ranging from 600 to 1000 ms. In the identity context, the correct interval (*t*_*c*_) was the same as *t*_*s*_, and in the remapped context, *t*_*c*_ was 1.5 times *t*_*s*_. In other words, the two contexts were identical during the measurement epoch but differed with respect to the production epoch. We denote these two contexts in terms of a gain factor relating *t*_*c*_ to *t*_*s*_: gain = 1 for the identity context, and gain = 1.5 for the remapped context. Subjects received trial-by-trial feedback about their performance (see Methods).

**Table 1:** Variables and abbreviations

MTN	Mental transformation noise
BIAS	Summary bias
√VAR	Summary standard deviation
RMSE	Root mean square error
*l*_*c*_	Correct length interval
*l*_*s*_	Sample length interval
*σ*_pre_	Pre-inference standard deviation
*σ*_post_	Post-inference standard deviation
*t*_*c*_	Correct time interval
*t*_*i*_	Inferred time interval
*t*_*m*_	Measured time interval
*t*_pre_	Pre-inference time interval
*t*_*p*_	Produced time interval
*t*_*s*_	Sample time interval
*θ*_c_	Correct angle
*θ*_s_	Sample angle
*w*_pre_	Pre-inference Weber fraction
*w*_post_	Post-inference Weber fraction
*f*_BLS_	Observer-actor Bayes-Least-Squares estimator
deg	Visual degrees
ms	Milliseconds
*H*_0_	Null hypothesis

We quantified performance with three statistics^8^: BIAS, which summarizes the deviation of average responses from the correct interval, √VAR, which summarizes the variability of responses across *t*_*s*_, and RMSE, which summarizes the total root mean square error. The three quantities are related through a sum of squares: RMSE^2^ = (√VAR)^2^ + BIAS^2^ (Fig. 1b). To ensure that the results were not influenced by overall tendencies to be late or early for all intervals, we calculated these statistics after removing an offset term that accounted for subjects’ overall bias (see Methods). For most subjects, the offset term was relatively small (see Supplementary Table 1).

Figure 2b illustrates the behavior of a typical subject in the identity (gray) and remapped (red) contexts. In the identity context, *t*_*p*_ had to match *t*_*s*_. However, responses were biased toward the mean of the prior distribution, consistent with Bayesian integration as was shown previously^8,26,27^ (Fig. 2b, in gray). In the remapped context, *t*_*p*_ had to be 50% longer than *t*_*s*_ (gain = 1.5). As expected by task requirements, *t*_*p*_ values were larger for each *t*_*s*_, and responses exhibited prior-dependent biases similar to the identity context.

**Fig. 3:**
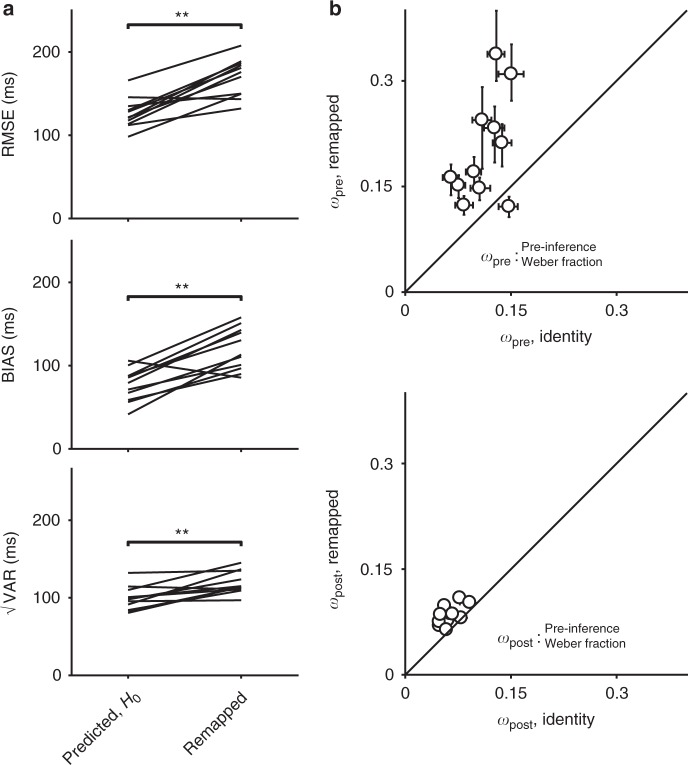
Summary of subjects’ behavior in the time measurement and production task, gain = 1.5. **a** Comparison of RMSE (top), BIAS (middle), and √VAR (bottom) for all subjects. The lines connect values predicted in the remapped context assuming no additional MTN (predicted, *H*_0_; left) based on the identity context (i.e., multiplied by the gain of 1.5) to actual values observed from behavior (gain = 1.5; right). Almost every subject had higher RMSE and BIAS than was predicted assuming no additional MTN. There was also a smaller increase in √VAR compared to predictions (**: *p* < 0.01, Wilcoxon two-sided signed-rank test). **b** Fitted model parameters. We fit an observer-actor model (see Methods) to each subject’s data independently for the two contexts. This model did not explicitly parameterize MTN, and so any differences in MTN across contexts were reflected in the Weber parameters *w*_pre_, capturing noise prior to inference and *w*_post_, capturing noise following inference. Most subjects were fit with much higher values of *w*_pre_ (top) in the remapped context, reflecting additional reliance on prior information consistent with a late inference strategy. There was also a more modest increase in *w*_post_ for the remapped context (bottom). Error bars represent 95% confidence intervals estimated using a bootstrap procedure (*n* = 1000). In the bottom panel, error bars are small and not visible (i.e., behind the open circle symbol)

We hypothesized that applying a gain of 1.5 would cause an increase in MTN, and would thus increase the total RMSE. Additionally, we hypothesized that the increase in RMSE would be predominantly due to an increase in bias, consistent with the late inference hypothesis. We tested the first hypothesis (increased MTN in the remapped context) by comparing behavior in the remapped context to that predicted under the null hypothesis of no additional MTN. The null hypothesis can be formulated straightforwardly by assuming that subjects multiplied estimates of *t*_*s*_ by 1.5. This predicts that the BIAS would be 1.5 times larger. Additionally, because of scalar variability in time production^28,29^, √VAR and RMSE would also have to be scaled by a factor of 1.5 (Fig. 2b, c). As shown by the example subject (Fig. 2c; predicted RMSE = 130 ms, 95% bootstrap CI = [126, 139] ms, *n* = 1000, actual RMSE = 187 ms, 95% CI = [176, 197] ms) as well as results across all 11 subjects (Fig. 3a, top), the observed RMSE in the remapped context was consistently and significantly higher than predicted under the null hypothesis (predicted RMSE median = 122 ms, interquartile range (IQR) = 19 ms vs. observed median = 176 ms, IQR = 36 ms, *p* = 0.002, Wilcoxon two-sided signed-rank test). This provides direct evidence that MTN increased in the remapped context and validates the logic of our experimental design in manipulating MTN via changing the gain independently of sensory noise.

Having established an increase in MTN in the remapped context, we tested the second hypothesis of whether the increase in RMSE was due to an increase in BIAS as predicted by the late inference strategy, or due to an increase in √VAR as predicted by the alternative early inference strategy (Fig. 1). As shown in Fig. 2c for one example subject, the increase in RMSE was associated with an increase in BIAS, which can be readily seen as an excess bias compared to the no additional MTN prediction (Fig. 2b; predicted BIAS = 86 ms, 95% CI = [79, 97] ms, *n* = 1000, actual BIAS = 140 ms, 95% CI = [128, 156] ms). The results for all subjects, summarized in Fig. 3a, indicate a clear increase in BIAS in the remapped context relative to what was expected under the hypothesis of no additional MTN (predicted BIAS median = 79 ms, IQR = 28 ms vs. observed median = 113 ms, IQR = 44 *p* = 0.002, Wilcoxon two-sided signed-rank test). Across subjects, there was also a smaller but consistent effect on √VAR (predicted median = 98 ms, IQR = 15 ms vs. observed median = 114 ms, IQR = 14 ms, *p* = 0.005, Wilcoxon two-sided signed-rank test). RMSE, BIAS, and √VAR for individual subjects are summarized in Supplementary Table 1. The substantial increase in BIAS rejects the early inference hypothesis and suggests that subjects implemented a late inference strategy.

Next, we compared the behavior of subjects in the two contexts using a Bayesian observer-actor model (Equation 4). This model comprises three stages: (1) a pre-inference stage characterizing all noisy processes before the inference, (2) a deterministic inference stage that uses knowledge about various sources of noise and statistical regularities to generate an estimate, and (3) a post-inference stage characterizing all noisy processes after the inference. We parameterized pre-inference and post-inference noise by Weber fractions *w*_pre_, and *w*_post_, respectively. The intervening inference stage implements an optimal Bayesian estimator, denoted *f*_BLS_, which minimizes the expected RMSE (Bayes least squares) given both pre- and post-inference sources of noise. The model established Bayes-optimal behavior in the identity context (Fig. 2b, “model fit,” bottom). We then fit the same model to subjects’ data in the remapped context, allowing *w*_pre_ and *w*_post_ to take different values (Fig. 2b, “model fit,” top). Since the model does not explicitly parameterize MTN, the effect of MTN has to be subsumed by *w*_pre_ and/or *w*_post_.

To compare early vs. late inference strategies quantitatively, we considered early inference and a late inference variants of the observer-actor model (Fig. 1a, middle and bottom). In the early inference model, *w*_pre_ was associated with sensory measurement, *f*_BLS_ acted after the sensory measurement and before the transformation, and *w*_post_ subsumed both MTN and production noise. In the late inference model, in contrast, *w*_pre_ subsumed measurement noise and MTN, *f*_BLS_ acted after the transformation and before production, and *w*_post_ was associated with production noise.

Before evaluating the model fits to subjects’ behavior in the two contexts, we used simulations to assess the behavior of the early and late inference models. As demonstrated in the Supplementary Figures 1, 2, in this model, increases in BIAS are associated with increases in *w*_pre_, and increases in √VAR, with increases in *w*_post_. Accordingly, if a subjects relies on the late inference strategy (i.e., larger BIAS), we would expect the model fits to yield larger value of *w*_pre_ in the remapped context compared to the identity context. In contrast, for the early inference strategy (larger √VAR), fits of the model should predominantly lead to higher values of *w*_post_. Simulations also revealed that BIAS of an observer-actor model that performs early inference is slightly larger for larger values of *w*_post_. However, this additional bias is qualitatively different from the bias predicted by late inference (i.e., increases in *w*_pre_). The bias caused by late inference is related to the integration of the prior and leads to a regression to the mean. In contrast, the bias increase of an observer-actor model in the early inference strategy is due to scalar variability and is independent of the prior^8,26^.

Given that the observed increase in BIAS in the remapped context was substantial, we predicted that fitting the model to the data would result in an increase in *w*_pre_ with little or no change in *w*_post_. Model fits supported this prediction: *w*_pre_ was substantially higher in the remapped context (median = 0.17, IQR = 0.09) compared to the identity context (median = 0.11, IQR = 0.05; *p* = 0.002, Wilcoxon two-sided signed-rank test; Fig. 3b, also see Supplementary Table 1). We also found a small increase in *w*_*post*_ (median = 0.09, IQR = 0.02 in the remapped context compared to median = 0.06, IQR = 0.01 in the identity context). These results substantiate that subjects used a late inference strategy to mitigate MTN, although the exact strategy employed by subjects may differ from the details of our model.

### Experiment 2: Ready, Set, Go with gain of  1 and 0.75

Another explanation that might account for the increased bias in the remapped context is that subjects did not learn the transformation correctly. One possibility is that, instead of applying a gain, subjects added a fixed offset to their responses for all sample intervals. Another possibility is that subjects did apply a gain, but their estimate of the gain was biased toward 1, which corresponds to the easier identity context. Both scenarios would result in an effective increase in bias and would therefore look qualitatively similar to the predictions of a late Bayesian inference strategy.

**Fig. 4:**
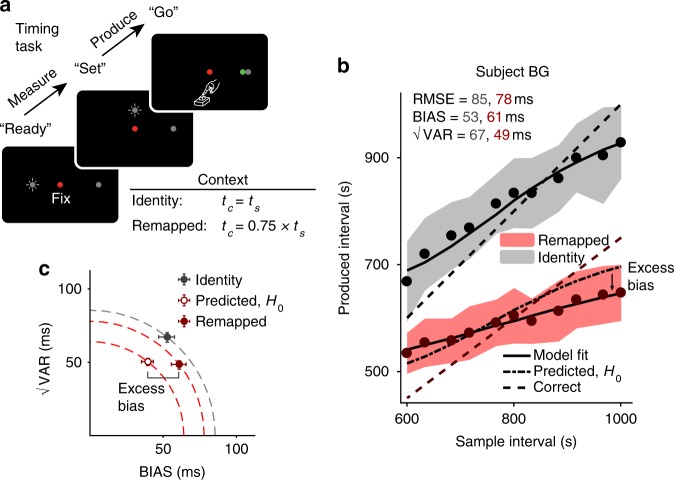
Time measurement and production task, gain =1 and  0.75. **a** Trial structure. Trial structure and feedback schedule were identical to that described in Fig. 2a, except that in the remapped context, the gain was 0.75. **b** Performance of an example subject in the identity (gray) and remapped (red) contexts. Results are shown in the same format as Fig. 2 except that the remapped data correspond to the gain of 0.75. Similar to gain = 1.5 (Fig. 2b), the subject’s behavior shows excess bias beyond what was predicted assuming no additional MTN. The same identity context data was used as a comparison for both gain = 0.75 and gain = 1.5. Shaded regions show mean ± one standard deviation. **c** √VAR vs. BIAS plot for the gain of 1 and 0.75 shown in the same format as Fig. 2c. RMSE in the and BIAS in the remapped context were both larger than predicted assuming no additional MTN. Error bars represent 95% confidence intervals estimated using a bootstrap procedure (*n* = 1000)

To rule out the two alternative explanations based on offset and biased gain, we devised another experiment in which subjects had to apply a gain of 0.75 (instead of 1.5) to their measurement of *t*_*s*_ (Fig. 4a). Importantly, for the gain of 0.75, the pattern of biases predicted by these alternative possibilities is the opposite of what is expected by late inference. If subjects were to use a fixed offset strategy to approximate a scaling with gain = 0.75, they should subtract a fixed interval from their responses. Doing so would result in overall shorter values of *t*_*p*_, but the slope of the relationship between *t*_*p*_ and *t*_*s*_ would be larger than expected from scaling responses by a factor of 0.75, resulting in smaller biases than predicted under the assumption of no additional MTN. Similarly, if subjects apply a biased gain (i.e., a gain that is closer to unity than 0.75), responses should be less biased than predicted from unity gain. These predictions can be readily compared with the predictions of the late inference model, which would predict the opposite pattern; i.e., increased biases similar to the gain of 1.5.

Figure 4b shows the behavior of one subject in the timing task for a gain factor of 0.75, compared with behavior in the unity context. Both RMSE and BIAS were larger in the remapped context (RMSE = 78 ms, 95% CI = [74, 82] ms; BIAS = 61 ms, 95% CI = [56, 66] ms) than predicted under the assumption of no additional MTN (RMSE = 64 ms, 95% CI = [60, 67] ms; BIAS = 40 ms, 95% CI = [36, 43] ms; Fig. 4c). In other words, results followed the same pattern that was observed for the gain of 1.5, suggesting that MTN was higher for the gain of 0.75 and subjects used late inference to account for it.

**Fig. 5:**
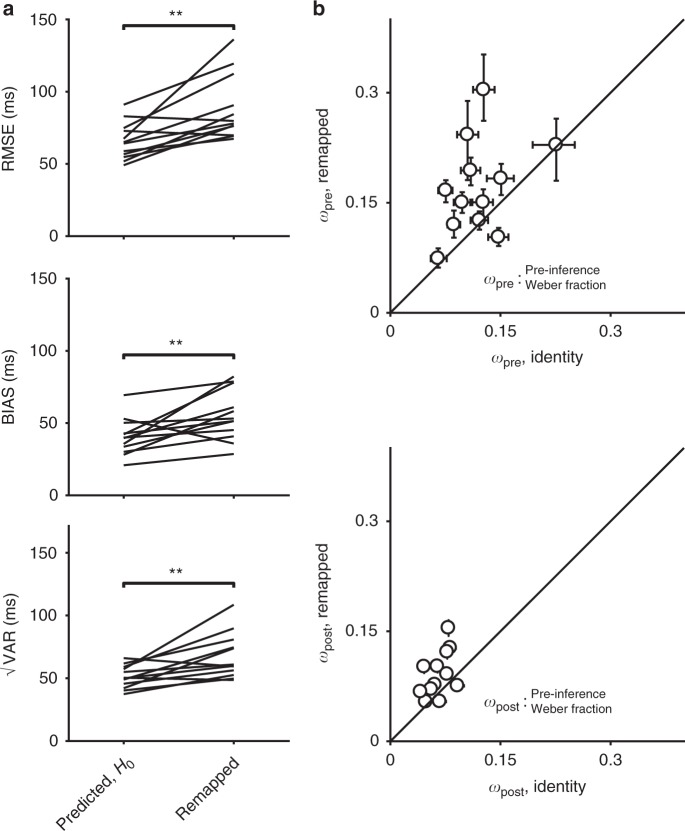
Summary of subjects’ behavior in the time measurement and production task, gain = 0.75. All results are shown using the same format as in Fig. 3 with the only exception that the remapped context corresponds to the gain of 0.75. **a** Comparison of RMSE (top), BIAS (middle), and √VAR (bottom) for all subjects. Most subjects had higher RMSE, BIAS, and √VAR than was predicted assuming no additional MTN (**: *p* < 0.01, Wilcoxon two-sided signed-rank test). The same identity context data was used as a comparison for both gain = 0.75 and gain = 1.5. **b** Fitted model parameters. Most subjects were fit with higher values of *w*_pre_ (top) in the remapped context, reflecting additional reliance on prior information consistent with a late inference strategy. There was also a more modest increase in *w*_post_ for the remapped context (bottom) for most subjects

Results for all subjects in the gain = 0.75 vs. identity context are shown in Fig. 5. Compared with values predicted by behavior in the identity context, subjects’ RMSE (predicted median = 65 ms, IQR = 18 ms vs. observed median = 79 ms, IQR = 29 ms, *p* = 0.002, Wilcoxon two-sided signed-rank test), BIAS (predicted median = 40 ms, IQR = 15 ms vs. observed median = 52 ms, IQR = 26 ms, *p* = 0.009, Wilcoxon two-sided signed-rank test), and fitted *w*_pre_ values were higher in the remapped context (identity median = 0.12, IQR = 0.04 vs. remapped median = 0.16, IQR = 0.09, *p* = 0.009, Wilcoxon two-sided signed-rank test).

One potential concern in this experiment is that applying a gain of 0.75 to the smallest values of *t*_*s*_ (e.g., 600 ms) requires subjects to produce intervals in the range of 450 ms, which demands relatively fast response times. Subjects whose natural reaction times are relatively slow may find it difficult to produce short *t*_*p*_ values, and their behavior may exhibit biases when *t*_*s*_ is short and gain is 0.75. To ensure the biases we observed were not due to such “floor effect”, we redid our analysis of BIAS for *t*_*s*_ greater than the median. Consistent with the full *t*_*s*_ distribution, BIAS was larger in the remapped context than predicted under the assumption of no additional MTN (observed median = 57 ms, IQR = 25 ms vs. predicted median = 44 ms, IQR = 15 ms, *p* = 0.007, Wilcoxon two-sided signed-rank test). See Supplementary Table 2 for individual subjects’ results. These results rule out the possibility that increased bias in the remapped condition can be explained by an offset or biased gain.

### Experiment 3: center-out reaching task with rotation of 0 and 60°

**Fig. 6:**
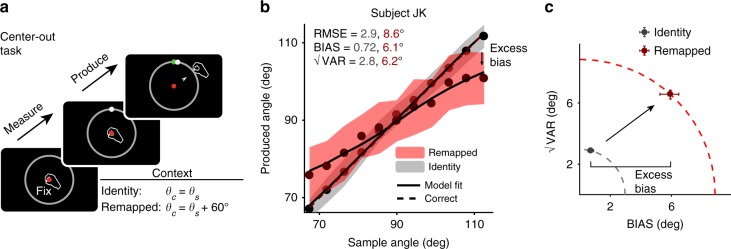
Center-out reaching task, visuomotor rotation = 0 and 60°. **a** Trial structure. While holding fixation on a central spot on an upwards facing monitor, subjects viewed a sample angle defined by the position of a small circle briefly flashed on a larger perimeter circle. Subjects then produced an angle by moving a handheld bar outward through the perimeter circle. In the “remapped” context (pictured), the response cursor was rotated 60° counterclockwise around the center of the circle relative to hand position such that correct angle *θ*_c_ = *θ*_s_ + 60°. The positions of the targets on the screen were identical across contexts. **b** Performance of an example subject in the identity (gray) and remapped (red) contexts (same format as Fig. 2). Similar to other tasks, the subject’s behavior showed excess bias beyond what was predicted assuming no additional MTN (i.e., equal to the identity context). Shaded regions show mean ± one standard deviation. **c** √VAR vs. BIAS plot shown in the same format as Fig. 2c. RMSE in the remapped context was larger than predicted assuming no additional MTN. Increased RMSE was due to increases in both BIAS (excess bias) and √VAR. Error bars represent 95% confidence intervals estimated using a bootstrap procedure (*n* = 1000)

So far, our results provide evidence that humans generate inferences by combining prior information with noisy mental transformations. However, is such inference specific to multiplicative temporal transformations or can it generalize across different types of transformations across modalities? To address this, we tested subjects on a center-out reaching task^19,30^ in which the transformation was not multiplicative but instead consisted of a constant visuomotor rotation^31^. Subjects viewed stimuli on an upwards facing monitor and then made responses by moving a digitizer beneath the monitor. Subjects were asked to move the cursor from a central starting position through the position of a target which was flashed briefly at the beginning of each trial at various angles on a visible circle (Fig. 6a). In the remapped context, the response cursor position was rotated 60° counterclockwise around the center of the circle relative to subjects’ hand position. Thus, this task also differed from the previous tasks in that the transformation was applied to subjects’ responses, rather than to the target values.

**Fig. 7:**
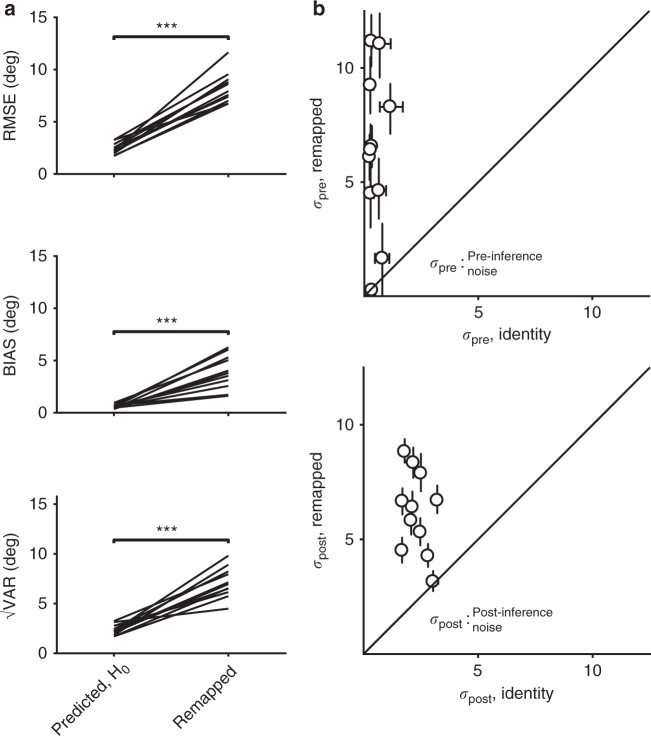
Summary of subjects’ performance in the center-out reaching task, visuomotor rotation = 60°. **a** Comparison of RMSE, BIAS, and √VAR for all subjects. On the left are values from individual subjects in the identity context; on the right are the values observed from behavior in the remapped context. Similar to the timing and length production tasks, subjects had higher error and were more biased towards the mean than was predicted assuming no MTN. Variability was also substantially increased in all subjects (***: *p* < 0.001, Wilcoxon two-sided signed-rank test). **b** Fitting the observer-actor model independently across the two contexts resulted higher values of σ_pre_ in the remapped context, reflecting additional reliance on prior information consistent with a late inference strategy. Values for σ_post_ were also substantially increased. Error bars represent 95% confidence intervals estimated using a bootstrap procedure (*n* = 1000)

Performance for one subject is shown in Fig. 6b, c, and population results are shown in Fig. 7. Because the transformation in this task did not involve a gain (scaling) factor, the no-MTN prediction for BIAS in the remapped context was equal to that measured in the identity context. Similarly, because in this task we modeled both measurement and production noise as non-scalar^1,2,30^, the no-MTN prediction for √VAR (and therefore also RMSE) was also equivalent to that computed in the identity context. Consistent with the tasks involving multiplicative transformations, both RMSE (8.6 deg, 95% CI = [8.2, 9.1] deg) and BIAS (6.1 deg, 95% CI = [5.6, 6.8] deg) were higher in the remapped context than in the identity context (2.9 deg, 95% CI = [2.7, 3.0] deg and 0.72 deg, 95% CI = [0.6, 0.9] deg, respectively), suggesting that MTN was larger for the transformation associated with a 60 degree visuomotor rotation. Across subjects, RMSE (identity median = 2.3, IQR = 0.76, remapped median = 7.9, IQR = 1.9, *p* = 0.001, Wilcoxon two-sided signed-rank test, *n* = 11 subjects), BIAS (identity median = 0.55, IQR = 0.29, remapped median = 3.8, IQR = 2.5, *p* = 0.001, Wilcoxon two-sided signed-rank test), and √VAR (identity median = 2.2, IQR = 0.78, remapped median = 6.96, IQR = 1.93, *p* = 0.001, Wilcoxon two-sided signed-rank test) were substantially higher in the remapped condition (Fig. 7a). Observer-actor model fits for the remapped context were associated with higher values of both pre-inference noise parameter *σ*_pre_ (identity median = 0.35, IQR = 0.39 deg vs. remapped median = 6.4, IQR = 4.5 deg, *p* = 0.002, Wilcoxon two-sided signed-rank test) and post-inference noise parameter *σ*_post_ (identity median = 2.2, IQR = 0.87 deg vs. remapped median = 6.4, IQR = 2.8 deg, *p* = 0.001, Wilcoxon two-sided signed-rank test; Fig. 7b). Supplementary Table 3 shows the parameter fits for individual subjects. The observed increase in bias and *σ*_pre_ in the remapped context indicate that applying a visuomotor transformation increased MTN associated with planning reach direction, and that subjects were able to deploy a late inference strategy to mitigate some of this additional noise, similar to other tasks. Additionally, the increase in variability observed in this task relative to previous tasks suggest that the transformation also generated some post-inference noise that subjects were not able to mitigate.

### Experiment 4: length measurement and identification task

**Fig. 8:**
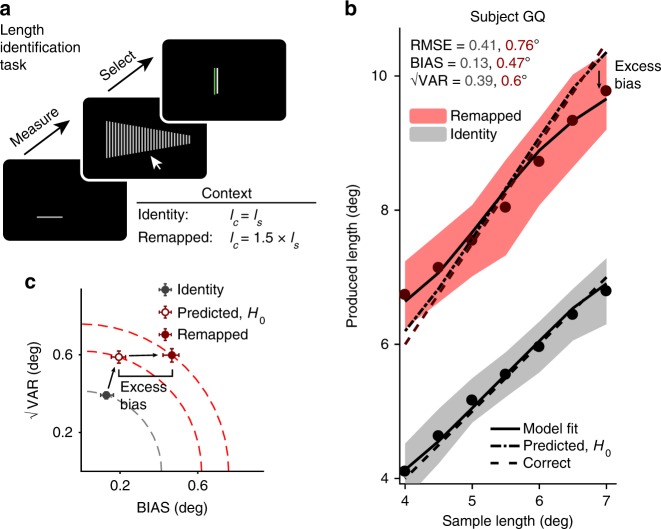
Length measurement and identification task, gain = 1 and 1.5. **a**Trial structure. Subjects first measured the sample length *l*_*s*_ of a gray horizontal bar presented briefly. After the bar was extinguished, subjects used the mouse to select from several options the bar which was as close in length as possible to the correct length *l*_*c*_ = gain × *l*_*s*_. In the identity context, gain = 1, whereas in the remapped context, gain = 1.5. There was no time limit to make a response. After the response, subjects were shown a white bar of the correct length, and the selected bar changed to either red or green depending on accuracy (see Methods). **b** Performance of an example subject in the identity (gray) and remapped (red) contexts (same format as Fig. 2). Similar to the sensorimotor tasks (Figs. 2b, 4b and 6b), the subject’s behavior showed excess bias beyond what was predicted assuming no additional MTN. Shaded regions show mean ± one standard deviation. **c** √VAR vs. BIAS plot shown in the same format as Fig. 2c. RMSE and BIAS in the remapped context were larger than predicted assuming no additional MTN. Error bars represent 95% confidence intervals estimated using a bootstrap procedure (*n* = 1000)

In experiments 1–3, we characterized the effect of noise in sensorimotor transformations during precise and rapid movements. However, humans can also perform mental transformations on sensory variables outside the domain of sensorimotor tasks. Therefore, we designed a task to test whether humans use late inference even when the inferred variable is perceptual and not directly involved in the control of a precise movement. Subjects had to perform a two-interval multiple-choice forced-choice task involving the measurement and identification of visual bars of different lengths (Fig. 8a). On each trial, subjects measured the length of a horizontal bar (i.e., sample length), and were subsequently presented with an array of vertical bars of different lengths. Subjects had to identify the bar whose length was either closest in length to the sample (identity context) or 1.5 times the sample (remapped context).

**Fig. 9:**
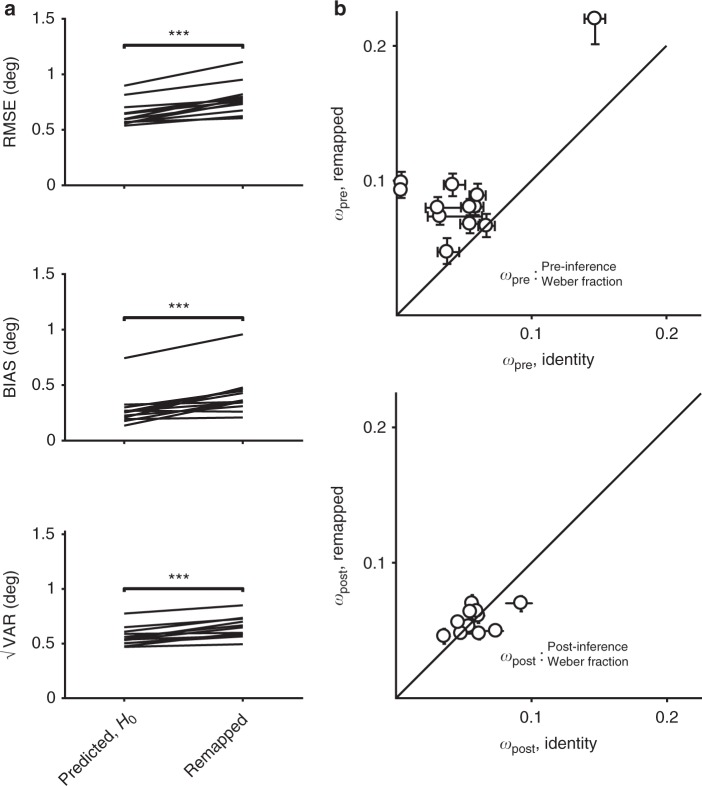
Summary of subjects’ performance in the length identification task, gain = 1.5. **a** Comparison of RMSE, BIAS, and √VAR for all subjects. On the left are values from individual subjects in the identity context; on the right are the values observed from behavior in the remapped context. Similar to the sensorimotor tasks, subjects had higher error and were more biased towards the mean and more variable than was predicted assuming no MTN (***: *p* < 0.001, Wilcoxon two-sided signed-rank test). **b**. Fitting the observer-actor model independently across the two contexts resulted higher values of *w*_pre_ in the remapped context, reflecting additional reliance on prior information consistent with a late inference strategy. Values for *w*_post_ were not significantly different between contexts. Error bars represent 95% confidence intervals estimated using a bootstrap procedure (*n* = 1000)

Figure 8b shows the behavior of an example subject. Because subjects were given as much time as needed to make their selections, we assumed that non-scalar execution noise of hand movements would not make a meaningful impact on behavior. Predictions for RMSE, BIAS, and √VAR in this task were therefore generated by multiplying values in the identity context by 1.5 as in experiments 1 and 2. Similar to the sensorimotor tasks, RMSE (0.76 deg, 95% CI = [0.71, 0.80] deg) for the example subject was larger in the remapped context than predicted (0.62 deg, 95% CI = [0.59, 0.65] deg). This result indicates that mental multiplication of a length by 1.5 is associated with higher MTN. Next we measured BIAS to assess whether performance was better explained by an early or late inference strategy. BIAS (0.47 deg, 95% CI = [0.41, 0.50] deg) was larger in the remapped context than predicted from the identity context (0.2 deg, 95% CI = [0.16, 0.24] deg, respectively) suggesting that the subject used the late inference strategy. These observations were consistent across subjects for both RMSE (predicted median = 0.62, IQR = 0.10, observed median = 81, IQR = 0.13, *p* = 0.0005, Wilcoxon two-sided signed-rank test, *n* = 12) and BIAS (predicted median = 0.21, IQR = 0.11, observed median = 0.37, IQR = 0.13, *p* = 0.0005, Wilcoxon two-sided signed-rank test; Fig. 9a). There was also an increase in √VAR across subjects (predicted median = 0.58, IQR = 0.10, remapped median = 0.70, IQR = 0.08, *p* = 0.001, Wilcoxon two-sided signed-rank test).

To model behavior in this experiment, we started with a measurement stage which was identical to that in experiments 1 and 2, reflecting the similarity in task structure. For the response stage, we simplified the model by replacing the task of choosing a response from a large (but finite) number of choices with the limiting case of choosing from an infinite number of choices. With this simplification, the model is mathematically equivalent to that used in experiments 1 and 2, parameterized by *w*_pre_ and *w*_post_. The observer-actor model fits for the remapped context were associated with higher values of *w*_pre_ (identity median = 0.05, IQR = 0.04 vs. remapped median = 0.09, IQR = 0.02, *p* = 0.005, Wilcoxon two-sided signed-rank test), while we found no systematic effect on *w*_post_ (identity median = 0.06, IQR = 0.01 vs. remapped median = 0.06, IQR = 0.02, *p* = 0.97, Wilcoxon two-sided signed-rank test; Fig. 9b). See Supplementary Table 4 for individual subjects’ results, and Supplementary Figure 4 for the results of a sensorimotor version of the task which were consistent with those shown here. These results indicate that the higher MTN in the remapped context is accompanied by higher reliance on prior information in perceptual as well as sensorimotor tasks.

## Discussion

The brain’s capacity to mitigate the effects of uncertainty was previously noted in experiments that focused on sensory and/or motor noise. The key advance in our work is the finding that the brain has the capacity to establish a late inference strategy so that behavior can account for the sources of noise associated with sensorimotor and mental transformations of sensory inputs.

As we found in our work, and others found in other behavioral settings^13,17–22^, mental transformations are noisy. Surprisingly however, most previous studies did not take this source of noise into consideration and focused on sensory inference^5,7,8,32^. While this approach may be adequate when the sensory noise is the dominant source of uncertainty^33^, mental transformations may generate a substantial fraction of the total noise for many tasks. Our experiment was designed to identify the effects of transformation noise explicitly by comparing identity and remapped behavioral contexts and thus manipulating MTN. However, many previous experiments that ignored MTN and yet found the behavior to be optimal might have misattributed MTN to noise in sensory representations. We also point out that while all of our experiments found evidence of a late inference strategy, subjects displayed increased variability in the remapped contexts beyond that predicted by an increase in pre-inference noise for most experiments. In this regard, one observation is particularly revealing: the experiment with the smallest increase in post-inference noise was the only purely perceptual task (experiment 4), and the experiment in which the transformation directly impacted movement-related visual feedback (experiment 3) was associated with the largest increase in post-inference noise. In the other experiments in which the transformation was between perceptual and motor domains, the increase in post-inference noise was intermediate. Based on these observations, we speculate that the extent to which MTN can be mitigated through late inference may be related to where along the sensory-to-motor pathways the transformation is implemented. It appears that the MTN is most effectively mitigated for mental transformations in the perceptual domain, less so for sensorimotor transformations and least for transformation that directly involve the motor system.

A late inference strategy would account for various observations in a wide range of tasks. One study found that reach movements in three-dimensional space were consistently biased towards the centroid of target distributions, particularly along the radial (distance) axis, and that this bias was not seen when subjects performed a simpler pointing task which only required wrist movements^17^. The authors’ interpretation of these results was that the brain implemented linear approximations to the true nonlinear transformations between target location and motor commands^34^. An intriguing alternative explanation might be that the increased bias was the result of additional uncertainty imposed by the more challenging task of reaching in 3D. Late inference may also explain patterns of biases in other tasks that involve complex coordinate transformations^35^, or in cases where the task involved complex and potentially noisy sensory computations^7^. Based on subjects’ performance in challenging tasks, a few groups have suggested that the brain uses knowledge of transformation noise to optimize behavior. For example, it has been shown that when both visual and proprioceptive information are available, subjects rely more strongly on the modality that had the least transformational complexity^21^. Schlicht and Schrater^13^ showed that subjects account for the effects of eye position uncertainty in a grasping task by increasing grip aperture. Moving beyond coordinate transformations, our late inference model provides a natural explanation for why various post-sensory cognitive operations may cause additional biases in behavior. Examples include mental operations in the presence of memory delays^36,37^, predictions of complex kinematics^38,39^, and pointing under various memory loads^20^. All these observations are consistent with our interpretation that humans have the capacity to mitigate noise associated with arbitrary transformations using late inference.

It is an open question how late inference might be implemented in the brain. In the standard formulation of Bayesian inference in the perceptual domain, sensory estimates are generated on a trial-by-trial basis by combining a likelihood function associated with an uncertain measurement with a prior distribution and a cost function. Implicit in this formulation is the assumption that observers can flexibly produce optimal inferences as one or more of these components change^40^. Similarly, late inference could involve a representation of transformation-associated likelihoods explicitly in the brain, such that observers can flexibly produce optimal behavior as task demands change. Ideally, the generation of intermediate (e.g., perceptual) point estimates (i.e., inferences) would be avoided such that the likelihood function used would encapsulate both sensory and MTN-related noise^25^.

Alternatively, subjects could learn to establish an optimal stimulus-to-response mapping by adjusting behavior based on trial-by-trial feedback without explicit representation of probabilistic quantities^41^. In this case, changes in the environment or internal noise structure would necessitate learning a new mapping. In our experiment, we did not verify whether subjects relied on a flexible model of MTN or a learned stimulus-to-response mapping. A more definitive assessment of how subjects learn to apply late Bayesian inference would likely require (1) experiments with multiple randomly intermixed gain factors and (2) assessment of performance in the absence of feedback. However, we speculate that the degree to which the brain can handle MTN flexibly might depend on familiarity with the task and the desired transformation. When the pertinent transformations are part of the daily behavioral repertoire, the brain may exhibit more flexibility and generalization. In contrast, when there is no need for generalization, optimization of behavior with respect to MTN may be more constrained. The levels of flexibility may also depend on the overlap between task demands and a participant’s skillset. For example, drummers, by virtue of their profession, may find it easier to apply sophisticated transformations to time intervals than others. While we do not know the various ways in which the brain learns to optimize behavior, our results indicate that a major goal of learning is to mitigate the effect of noise in mental transformations.

## Methods

### Subjects

Human subjects aged 18–65 years participated in this study after giving informed consent. All experiments were approved by the Committee on the Use of Humans as Experimental Subjects at the Massachusetts Institute of Technology. Semi-overlapping groups of 11 or 12 subjects completed experiments 1 and 3, and experiments 2 and 4, respectively. Eleven subjects completed an additional experiment shown in Supplementary Figure 4. Differences in number of subjects across tasks were due to subject attrition. All subjects had normal or corrected-to-normal vision.

### Procedures

Experimental sessions lasted ~45–60 min. Each subject completed 1–4 sessions per week. Experiments were controlled by an open-source software (MWorks; http://mworks-project.org/). All trials in experiments 1–3 began with central fixation spot on a black background that subjects were asked to hold their gaze on throughout the trial. Eye movements were not monitored. In experiments 1, 2, and 4, subjects viewed all stimuli binocularly from a distance of ~67 cm on either a 23-inch Apple A1082 LCD monitor at a resolution of 1900 × 1200 driven by an Intel Macintosh Mac Pro computer, or a 24-inch early 2009 Apple Mac Pro at a refresh rate of 60 Hz in a dark, quiet room. In experiments 1 and 2, responses were registered on a standard Apple Keyboard connected to the experimental machine. In experiment 3 as well as the experiment in Supplementary Figure 4, subjects viewed stimuli from above on a 21.5-inch Samsung SyncMaster SA200 monitor, and responses were registered using a pen digitizer tablet (Wacom Intuos5 touch); the stylus was fixed at a vertical position inside a custom printed handle which subjects grasped. In experiment 4, responses were registered with a standard computer mouse.

### Behavioral tasks

Our objective in all experiments was to investigate the effect of mental transformation noise (MTN) on performance. To do so, each experiment consistent of two contexts, an “identity” context, and a more challenging “remapped” context that was expected to involve higher levels of MTN. In each experiment, human subjects measured a scalar sensory quantity (time interval, length, or angle on a circle) drawn from a prior distribution. In the “identity” context, subjects had to reproduce or identify the sensory quantity (the sample), and in the “remapped” context, they had to produce or identify the same quantity subjected to a linear transformation. Subjects whose responses for the shortest and longest stimuli in the identity context were at least one d′ (d-prime) apart were invited to participate in the main experiment. Three subjects were excluded, all for experiments 1 and 2. Subjects completed one training session for each experimental condition to familiarize with the task and verify that their behavior was reasonably stable. These training sessions were followed by two to three test sessions on which the reported analyses were conducted.

### Experiments 1 and 2: time interval estimation and production task

The behavioral task used in experiment 1 was a variant of the Ready, Set, Go task used in a previous study^8^. Subjects had to measure a sample interval drawn from an 11-point discrete uniform distribution between 600 and 1000 ms, then immediately produce an interval that was equal to the sample interval multiplied by a gain factor. The sample interval was demarcated by two visual flashes (“Ready” and “Set”) located to the left and above a fixation point at the center of a computer monitor. The production interval was defined as the interval between the onset of the second flash and the response (key press) of the subject. In the identity context, the gain was 1, whereas in the remapped contexts the gain was either 1.5 or 0.75. The gain was fixed in each behavioral session and was communicated at the beginning of each session as either “same,” “shorter,” or “longer.” The gain was also provided visually: the ratio between the distances of a “Go” target and Ready to the fixation point was equal to the gain factor.

### Feedback schedule

Following each response, subjects were informed about their performance by a circular feedback stimulus which appeared either to the left (for early responses) or right (for late responses) of the Go target. The distance between the feedback stimulus and the Go target was proportional to the magnitude of the error, and the color of the feedback stimulus was either green to indicate a “hit” or white to indicate a “miss.” Subjects completed two consecutive sessions of 600 trials for the identity context; the hit/miss threshold was on a trial-by-trial one-up one-down staircase for the first training session and fixed for the second test session at the mean of the last 100 trials of the first session. For the remapped context sessions (also 600 trials), the error threshold was on a one-up one-down staircase. Classifying trials as hits or misses only served as motivation for subjects and was not pertinent to the analyses nor did it affect the task contingencies beyond the color of the feedback stimulus.

### Experiment 3: center out reaching task

Subjects were briefly presented (500 ms duration) with a target placed on a response circle of approximately 10° radius with an angle drawn from an 11-point discrete uniform distribution 45 degrees wide and centered at directly ahead. Subjects were instructed to then rapidly reach “through” the target location in a single straight movement. Once the response motion had commenced, as measured by the manipulandum crossing an eccentricity threshold of 2° from the starting position at the center of the response circle, a small circle tracked the position of the manipulandum. The “production” was the angle at which the cursor intersected the response circle. To encourage ballistic movements and minimize online error correction, subjects had 250 ms to reach the perimeter of the response circle once movement was detected. In this task, the transformation was applied to the response rather than the sample: in the remapped context, the position of the manipulandum indicated on the screen, as well as the produced angle, was rotated 60° clockwise. The transformation was communicated by telling subjects that they should proactively correct for an imposed rotation, but the exact angle of the rotation was not given. Response feedback was similar to the previous two tasks, with the difference being that the feedback was rotated according to the context, with which subjects could learn the angle of rotation. The produced angle was shown as a green or red disk placed on the response perimeter along with the correct target position. As in the length measurement and production task, subjects completed four sessions total with each session comprising two blocks of 150 trials of identity and remapped trials. We excluded the first 25 trials of each block from analysis to reduce the impact of transient adaptation effects^31^. The error threshold for each gain was on a staircase for the first two sessions and fixed for the final two at the mean of the last 100 trials for each gain. The order of identity and remapped blocks (identity first or second) was counterbalanced across subjects.

### Experiment 4: length measurement and identification task

Subjects measured a briefly presented (50 ms duration) horizontal gray line with length drawn from a 7-point discrete uniform distribution between 4 and 7 degrees visual angle. The position of the sample line was randomly selected within a 6 by 6 degrees visual angle window centered on the screen. After the horizontal line was extinguished and a delay of 500 ms, subjects were shown a set of 29 vertical lines of different lengths and selected the response line using a mouse. There was no time limit to make a response. In the identity context subjects were asked to select the line matching the length of the sample line with response lines evenly spaced between 2 and 9 degrees visual angle, while in the remapped context subjects selected a response line 1.5 times the length of the sample with response lines evenly spaced between 3 and 13.5 degrees visual angle. The order of the response lines, ascending or descending from the left side of the screen, was randomly determined across trials. The vertical location of the response lines was randomly positioned within 3 degrees visual angle from the center of the screen. The gain was communicated by telling subjects at the start of the first session to produce either “the same as” or “one and a half times” the length of the sample. For feedback, the correct length was shown in white to the right of the selected line which was shown in green for a “hit” if the selected line was equal to or within one choice of the correct length and red for a “miss” otherwise. As in experiment 4, subjects completed four sessions with two blocks of 150 trials for each context. The order of blocks was counterbalanced across subjects.

### Data analysis

Behavioral performance in all tasks was quantified with three statistics^8^: BIAS, √VAR, and RMSE. BIAS summarizes the difference between average and correct responses across all sample values and is defined for the case of a discrete uniform prior distribution as

1 $${\mathrm{BIAS}} = \sqrt {E[{\mathrm{bias}}^2]} = \sqrt {\frac{1}{N}\mathop {\sum }\limits_{i = 1}^N {\mathrm{bias}}_i^2}$$

where bias_*i*_ is the difference between the mean response and correct response for a particular sample value and *N* is the number of sample values tested. √VAR summarizes the variability of responses:

2 $$\sqrt {{\mathrm{VAR}}} = \sqrt {E[{\mathrm{var}}]} = \sqrt {\frac{1}{N}\mathop {\sum }\limits_{i = 1}^N {\mathrm{var}}_i}$$

where var_*i*_ is the variance of the responses for a particular sample value. Because samples were drawn randomly, it was not the case that the number of trials for each sample was exactly the same. Therefore, averages of for BIAS and √VAR were normalized across samples according to the number of trials presented. Finally, RMSE was calculated as:

3 $${\mathrm{RMSE}} = \sqrt {{\mathrm{VAR}} + {\mathrm{BIAS}}^2}$$

Prior to analyzing data, we identified and removed “lapse” trials for each subject. This involved finding and removing trials for which responses were greater than three times the median absolute deviation from the mean response for a particular sample quantity and context. All confidence intervals were basic bootstrap confidence intervals (*n* = 1000).

### Model descriptions and fitting procedure

Previously, we found that a Bayesian observer model captured the behavior of human subjects in the RSG task, explaining behavior explicitly in terms of noise associated with sensory measurement and motor production^8^. Here, the model is formulated more generally in terms of pre- and post-inference processes, as we are interested in identifying additional noisy computations beyond measurement and production which may occur prior to or after inference. Additionally, following previous work showing that humans account for response variability when planning actions^9,10^, we augmented the inference stage to account for post-inference variability^26^. This “observer-actor” model consists of three stages: a noisy pre-inference stage, a deterministic Bayesian inference stage, and a noisy post-inference stage. We describe the model for the RSG task, although the structure is the same for all experiments. First, the noisy pre-inference value *t*_pre_ (*t* represents time) is generated according to the noise model *p*(*t*_pre_|*t*_*s*_), then used to generate an inference *t*_*i*_ which minimizes the expected loss given *t*_pre_:

4 $$t_i = f_{{\mathrm{BLS}}}\left( {t_{{\mathrm{pre}}}} \right) = \arg \mathop {{\min }}\limits_t \mathop {\smallint }\limits_{t_c}^{} \mathop {\smallint }\limits_{t_p}^{} \left( {t_p - t_c} \right)^2p(t_c|t_{{\mathrm{pre}}})p(t_p|t)\pi (t_c){\mathrm d}t_p{\mathrm d}t_c$$

In this equation, π(*t*_*c*_) represents the observer’s “prior” belief about the correct value being inferred, *t*_*c*_, and the loss function is the squared difference between *t*_*p*_ and *t*_*c*_ (squared error). Note that *t*_pre_ can represent either the noisy measurement or the noisy transformed value, and π(*t*_*c*_) can represent the prior over sensory or transformed values, depending on whether the observer adopts an early or late inference strategy, respectively (Fig. 1a). The model then generates *t*_*p*_ according to the production noise model *p*(*t*_*p*_|*t*_*i*_). The observer-actor model has two free parameters which are associated with the variances of *p*(*t*_pre_|*t*_*s*_) and *p*(*t*_*p*_|*t*_*i*_). For RSG (experiments 1 and 2), both *p*(*t*_pre_|*t*_*s*_) and p(*t*_*p*_|*t*_*i*_) were modeled as scalar Gaussian random variables (i.e., with standard deviation proportional to the mean) with parameters *w*_pre_ and *w*_post_ representing the Weber fractions for *p*(*t*_pre_|*t*_*s*_) and *p*(*t*_*p*_|*t*_*i*_), respectively. In experiment 3, both measurement and production were modeled as Gaussian random variables *σ*_pre_ and *σ*_post_, while in experiment 4, both measurement stages were modeled as scalar Gaussian random variables.

The solution to Eq. 4 depends on the model for production noise. For the case of Gaussian production noise (experiment 3), the optimal inference is the conditional expectation of *t*_*c*_ given *t*_pre_, or $t_i = E[t_c|t_{{\mathrm{pre}}}]$. For scalar Gaussian noise (experiments 1, 2, and 4), $t_i = E\left[ {t_c{\mathrm{|}}t_{{\mathrm{pre}}}} \right]/(1 + w_{{\mathrm{post}}}^2)$. The denominator modestly shifts inferences towards shorter values to avoid the increased noise associated with longer values under scalar variability. However, this effect on BIAS is small compared to that associated with the prior. Generally for all experiments, the model captures high response variability by increases in *w*_post_, and large response biases toward the mean of the prior by increases in *w*_pre_. See Supplementary Figures 1 for a simulation of the effects of *w*_pre_, and *w*_post_, and Supplementary Figure 2 and Supplementary Methods for a formal analysis in the case of Gaussian internal noise and prior.

Model parameters were fit by maximizing the log-likelihood of subjects’ responses given the sample values and gain. The maximization was done using the fminsearch command in MATLAB (Mathworks). Model fitting and simulation involved numerical integration over the posterior distribution using Simpson’s rule. Parameter searches were repeated ten times each with different parameter initialization, and results were inspected for consistency.

## Electronic supplementary material

Supplementary Information

## Data Availability

The datasets generated during and/or analyzed during the current study are available from the corresponding author on reasonable request.
