# Supplementary material for: Late Bayesian inference in mental transformations
Source: Nat Commun. 2018 Oct 24;9:4419. doi: 10.1038/s41467-018-06726-9 (PMC6200789; doi:10.1038/s41467-018-06726-9)
Supplement: Supplementary file 1 — Supplementary Information [file 41467_2018_6726_MOESM1_ESM.pdf]

# Supplemental Information

# Late Bayesian inference in mental transformations

Evan Remington, Tiffany V. Parks, Mehrdad Jazayeri

## Supplementary Figures

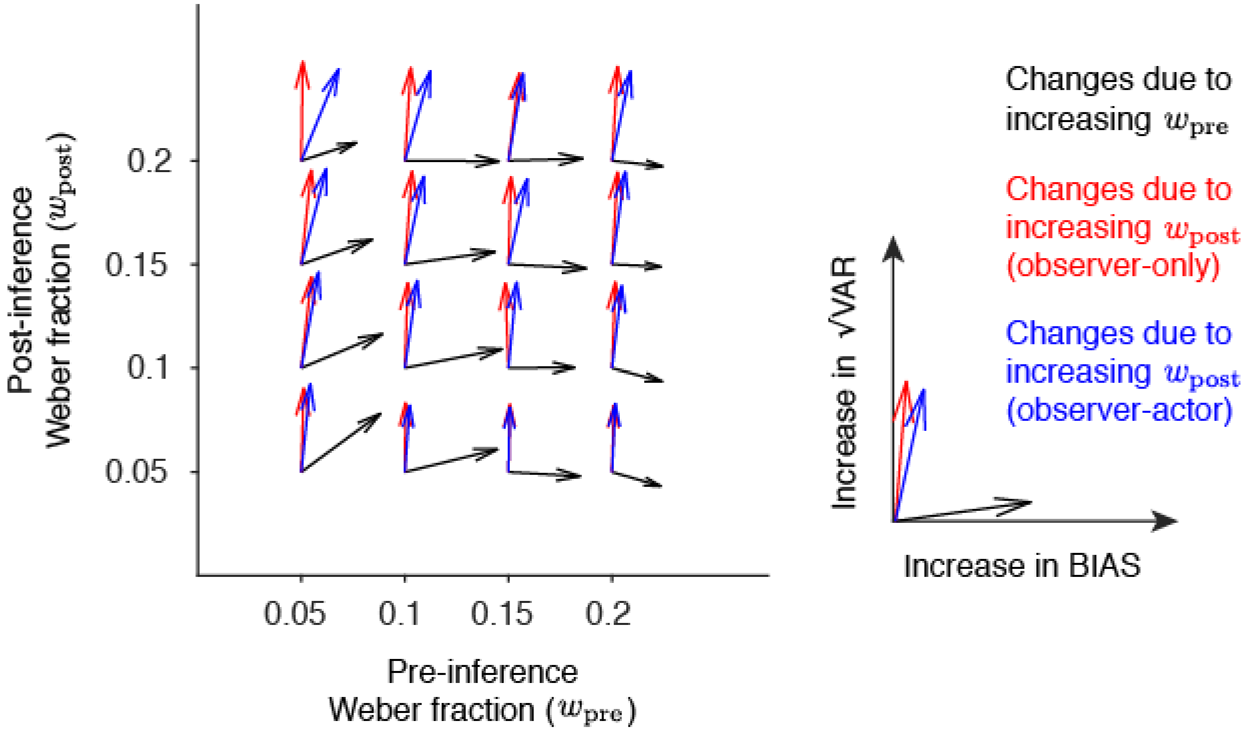


**Supplementary Figure 1.** Simulation of effects of pre- and post-inference noise on bias and variance. Our reasoning that increases in bias constitute evidence for a late-inference process in the brain hinges on two propositions for a Bayes least squares observer which minimizes root mean square error (RMSE). These propositions are that, (1) increases in pre-inference noise, such as that due to sensory measurements, generally increase bias, whereas (2) increases in post-inference noise, such as that due to motor execution, primarily result in increases in variance. Because it is not straightforward to solve the minimization of the loss function described in equation 4 for scalar measurement noise or a discrete uniform prior distribution, we illustrated the effects of pre- and post-inference noise on BIAS and √VAR through simulation. Using the Bayesian observer-actor model, we simulated the effects of varying scalar pre-inference noise (black: $w_{pre}$: pre-inference Weber fraction) and post-inference noise (blue; $w_{post}$: production Weber fraction) on BIAS and √VAR. For comparison, we also simulated the effects of $w_{post}$ in the observer model [1] (red, "observer-only") which does not take post-inference noise into account when generating inferences. For both models, increasing the value of $w_{post}$ substantially increased variability with a comparably small increase in bias, whereas increasing $w_{pre}$ increased bias but had little effect on variability. BIAS and √VAR were calculated by averaging over many simulations, each with a similar number of trials completed by subjects in experiments 1 and 2. We note that a small amount of increase in bias due to $w_{post}$ in the simulations can be attributed to sampling noise due to the finite number of trials. Thus, we interpret increases in $w_{pre}$ for subjects in the remapped contexts as evidence for reliance on prior information in a late inference strategy to mitigate the effects of increased MTN relative to the identity contexts.

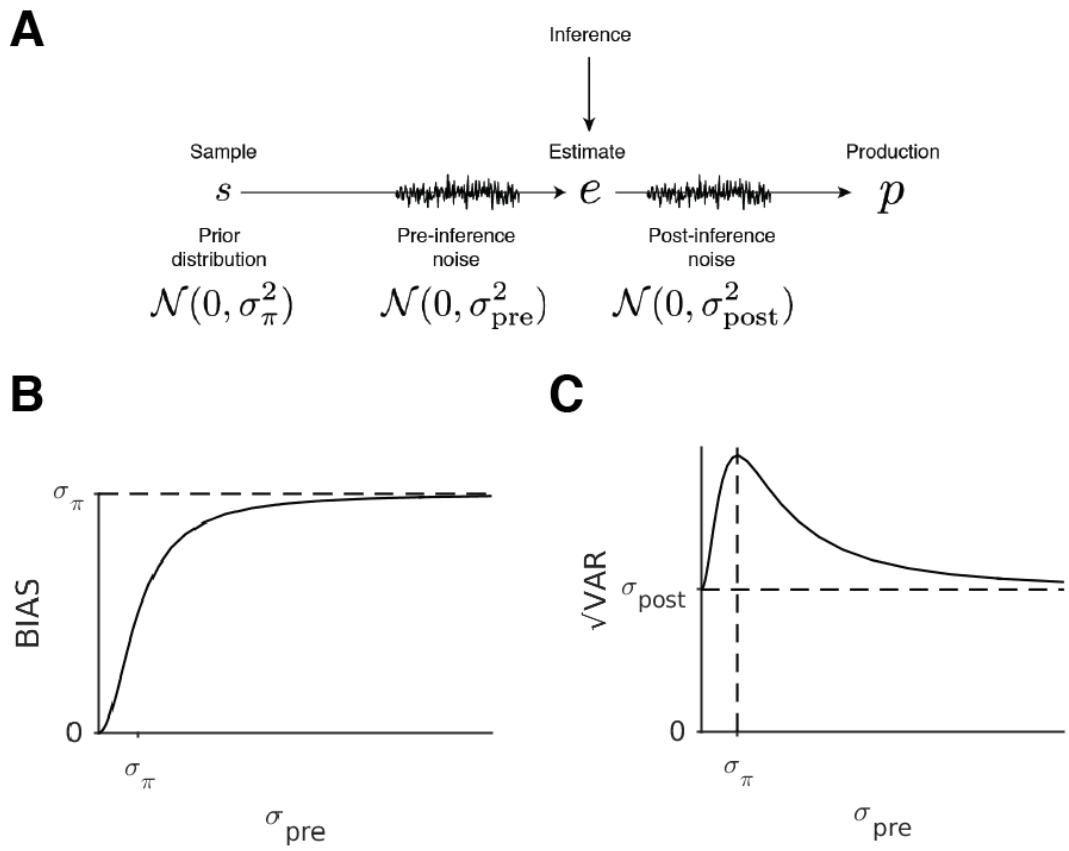


**Supplementary Figure 2.** Effects of pre- and post-inference noise on bias and variance for Gaussian Noise. (**A**). Model schematic. In the case of Gaussian noise and Gaussian prior, solutions of both BIAS and √VAR can be derived. The sample is drawn from a zero-mean Gaussian distribution with standard deviation, $\sigma_\pi$, and the pre- and post-inference noise have zero-mean Gaussian distributions with standard deviations $\sigma_{\mathrm{pre}}$ and $\sigma_{\mathrm{post}}$, respectively. For this scenario, the BIAS and √VAR can be analytically computed (see **Supplementary Methods**). (**B**). BIAS vs. $\sigma_{\mathrm{pre}}$. BIAS increases monotonically increases as a function of $\sigma_{\mathrm{pre}}$, asymptotically approaching $\sigma_\pi$ when $\sigma_{\mathrm{pre}}^2 >> \sigma_\pi^2$. BIAS does not depend on $\sigma_{\mathrm{post}}$ (**Supplementary Methods**). (**C**). √VAR vs. $\sigma_{\mathrm{pre}}$. For values of $\sigma_{\mathrm{pre}}$ close to 0, increases in $\sigma_{\mathrm{pre}}$ have a substantial impact on √VAR. However, increases in $\sigma_{\mathrm{pre}}$ actual results in decreases in √VAR when $\sigma_{\mathrm{pre}} > \sigma_\pi$. For values of $w_{\mathrm{pre}}$ or $\sigma_{\mathrm{pre}}$ fit to most subjects in our experiments, increases in pre-inference noise contributed primarily to increases in BIAS.

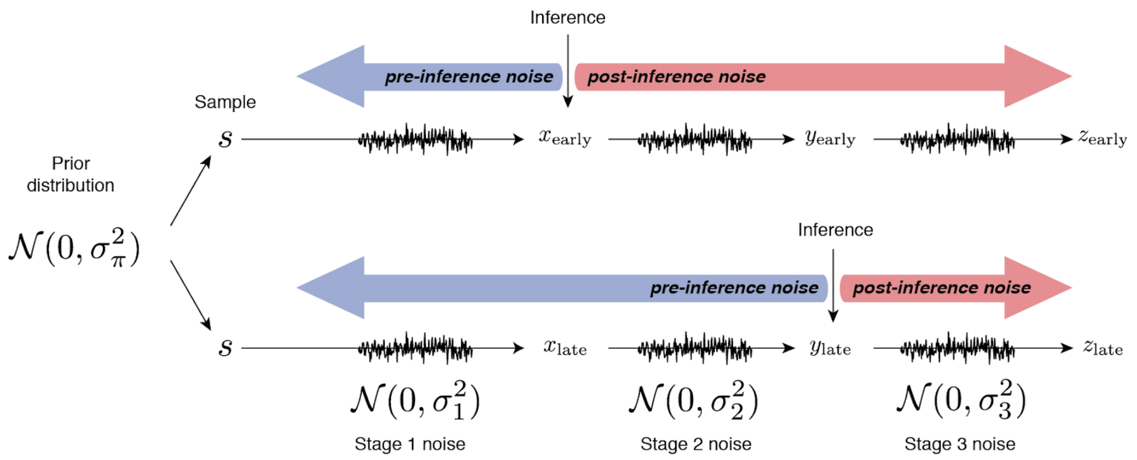


**Supplementary Figure 3.** Models for analytical comparison of early and late inference with Gaussian prior and Gaussian noise. The superiority of the late inference over early inference can be shown analytically if we assume that the prior and noise have Gaussian distributions. Consider that the sample, *s*, is drawn from a zero-mean prior with standard deviation of $\sigma_\pi$. The generation of output goes through three noisy computational stages that sequentially convert *s* to *x*, *x* to *y* and *y* to *z*. We will use subscript "early" or "late" for the variables to distinguish between these two strategies. The early inference strategy uses the prior to infer the value of *s* after the first computational stage ($x_{\text{early}}$). In this case the noise in stage 1 acts as pre-inference noise, and the inferred value is perturbed subsequently by two post-inference sources of noise to compute $y_{\text{early}}$ and $z_{\text{early}}$. In the late inference strategy, noise in the first two stages act as pre-inference noise, inference is made at the second stage to generate $y_{\text{late}}$, which is then perturbed by the post-inference noise in stage 3 resulting in $z_{\text{late}}$. See **Supplementary Methods** for derivation.

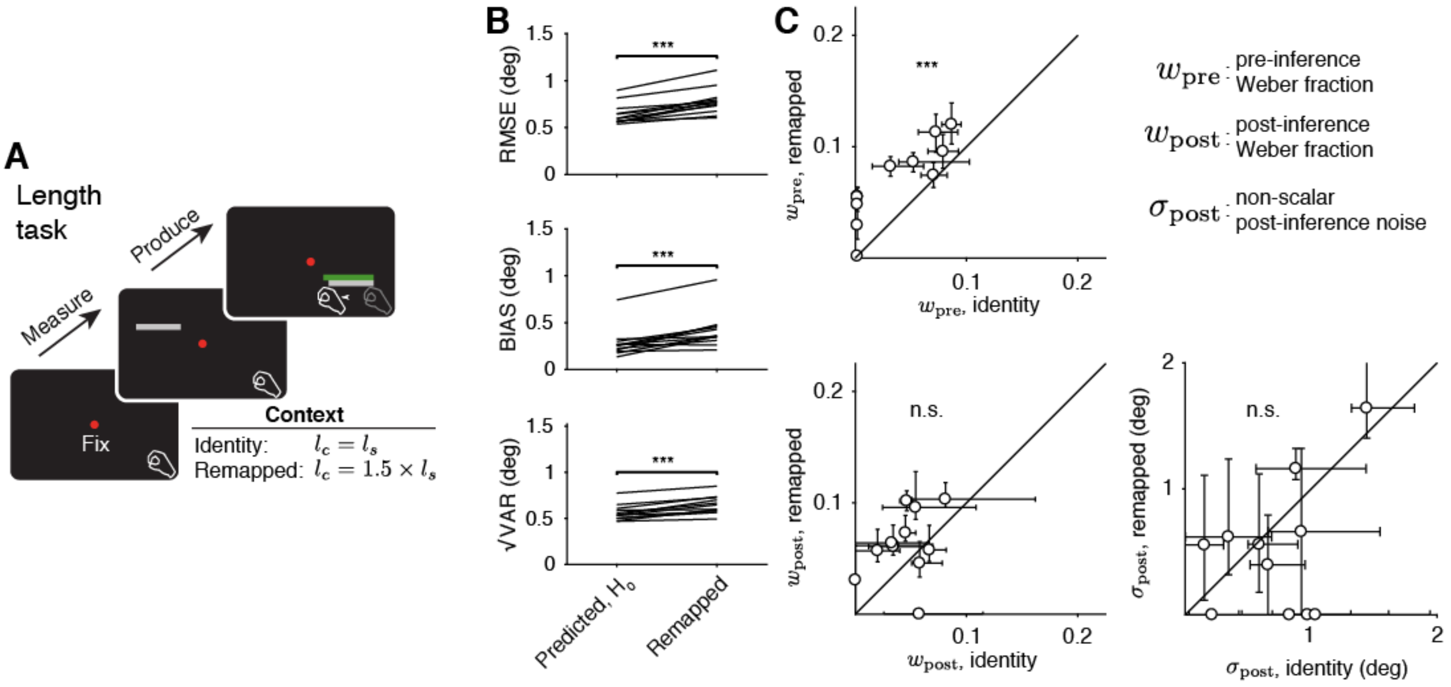


**Supplementary Figure 4**. Length measurement and production task. **A**. Trial structure. Each trial began with the presentation of a red fixation spot. Subjects first measured the sample length $l_s$ of a gray bar presented briefly on an upwards facing monitor. After the bar was extinguished, subjects moved a manipulandum containing a digitizing pen located under the monitor in order to draw a bar which was as close in length as possible to the correct length $l_c$ = gain x $l_s$. In the identity context, gain = 1, whereas in the remapped context, gain = 1.5. After the response, subjects were shown a gray bar of the correct length, and the produced bar changed to either red or green depending on accuracy (see **Supplementary Methods**). (B). Comparison of RMSE (top), BIAS (middle), and √VAR (bottom) for all subjects in the length production task. All results are shown using the same format as in Figure 3. Almost every subject had higher RMSE and BIAS than was predicted assuming no additional MTN. Variability also increased in most subjects (***: $p < 0.001$, Wilcoxon two-sided signed-rank test; n = 11 subjects). (C) Fitted model parameters. Most subjects were fit with higher values of $w_{pre}$ (top) in the remapped context, reflecting additional reliance on prior information consistent with a late inference strategy. Values for $w_{post}$ and the non-scalar noise parameter $\sigma_{post}$ were not systematically affected by the gain (n.s.; not significant). We note that although this task involved a memory delay, because the memory load was present in both contexts, our experiment affords a direct comparison of the effect of sensorimotor noise across the conditions. These results indicate that the higher MTN in the remapped context is accompanied by higher reliance on prior information, as expected from a late inference strategy. Error bars represent 95% confidence intervals estimated using a bootstrap procedure (n = 1000).

## Supplementary Methods

### Bias and variance for Gaussian Noise

For Gaussian noise and prior distribution (**Supplementary Figure 2A**), the BIAS can be analytically computed as follows:

$$\mathrm{BIAS} = \sqrt{\mathrm{E}[\mathrm{bias}^2]} = \frac{\sigma_{\mathrm{pre}}^2 \sigma_\pi}{\sigma_{\mathrm{pre}}^2 + \sigma_\pi^2} \qquad \text{(S1)}$$

Evidently, the BIAS only depends on $\sigma_{\text{pre}}$, and not $\sigma_{\text{post}}$. This validates our assertion that changes of pre-inference noise are reflected in changes BIAS. This formulation additionally reveals that when $\sigma_{\mathrm{pre}}^2 >> \sigma_\pi^2$, BIAS asymptotically reaches $\sigma_\pi$ (**Supplementary Figure 2B**).

√VAR can also be analytically computed as follows:

$$\sqrt{\mathrm{VAR}} = \sqrt{\mathrm{E}[\mathrm{var}]} = \sqrt{\left(\frac{\sigma_{\mathrm{pre}}}{1 + \sigma_{\mathrm{pre}}^2/\sigma_\pi^2}\right)^2 + \sigma_{\mathrm{post}}^2} \qquad \text{(S2)}$$

The effect of $\sigma_{\text{post}}$ only appears in equation S2, indicating that $\sigma_{\text{post}}$ can only influence √VAR. This validates our assertion that changes of post-inference noise only impact √VAR. Interestingly, the relative effect of increasing $\sigma_{\text{pre}}$ on √VAR depends on the relative values of $\sigma_{\text{pre}}$ and $\sigma_\pi$, (**Supplementary Figure 2C**). For values of $\sigma_{\text{pre}}$ close to 0, increases in $\sigma_{\text{pre}}$ have a substantial impact on √VAR. However, for larger values of $\sigma_{\text{pre}}$ relative to $\sigma_\pi$, $\sigma_{\text{pre}}$-related changes diminish and then become negative when $\sigma_{\text{pre}} > \sigma_\pi$. Another way to see this is to take the derivative of VAR with respect to $\sigma_{\text{pre}}$:

$$\frac{d(\mathrm{VAR})}{d\sigma_{\mathrm{pre}}} = \frac{2\sigma_{\mathrm{pre}}}{1 + \sigma_{\mathrm{pre}}^2/\sigma_\pi^2} * \frac{1 - \sigma_{\mathrm{pre}}^2/\sigma_\pi^2}{\left(1 + \sigma_{\mathrm{pre}}^2/\sigma_\pi^2\right)^2} \qquad \text{(S3)}$$

The term $1 - \sigma_{\mathrm{pre}}^2/\sigma_\pi^2$ in Equation S3 indicates that (1) increasing $\sigma_{\text{pre}}$ beyond $\sigma_\pi$ reduced VAR (and therefore √VAR) and (2) the global maximum of √VAR corresponds to $\sigma_{\text{pre}} = \sigma_\pi$. Inspecting S2 directly, the net contribution of $\sigma_{\text{pre}}$ to √VAR becomes 0 when $\sigma_{\text{pre}} \gg \sigma_\pi$.

### Analytical comparison of early and late inference with Gaussian prior and Gaussian noise.

We can analytically compute the BIAS, √VAR and RMSE for the early and late inference strategies (see **Supplementary Figure 3**) as follows:

$$\text{BIAS}_{\text{early}} = \frac{\sigma_\pi \sigma_1^2}{\sigma_1^2 + \sigma_\pi^2} \tag{S4}$$

$$\sqrt{\text{VAR}}_{\text{early}} = \sqrt{\frac{\sigma_\pi^4 \sigma_1^2}{(\sigma_1^2 + \sigma_\pi^2)^2} + \sigma_2^2 + \sigma_3^2} \tag{S5}$$

$$\text{RMSE}_{\text{early}} = \sqrt{\frac{\sigma_\pi^2 \sigma_1^2}{\sigma_1^2 + \sigma_\pi^2} + \sigma_2^2 + \sigma_3^2} \tag{S6}$$

$$\text{BIAS}_{\text{late}} = \frac{\sigma_\pi (\sigma_1^2 + \sigma_2^2)}{\sigma_1^2 + \sigma_2^2 + \sigma_\pi^2} \tag{S7}$$

$$\sqrt{\text{VAR}}_{\text{late}} = \sqrt{\frac{\sigma_\pi^4 (\sigma_1^2 + \sigma_2^2)}{(\sigma_1^2 + \sigma_2^2 + \sigma_\pi^2)^2} + \sigma_3^2} \tag{S8}$$

$$\text{RMSE}_{\text{late}} = \sqrt{\frac{\sigma_\pi^2 (\sigma_1^2 + \sigma_2^2)}{\sigma_1^2 + \sigma_2^2 + \sigma_\pi^2} + \sigma_3^2} \tag{S9}$$

We can directly compare the performance of these two strategies by comparing the corresponding mean-squared-errors (MSE):

$$\Delta\text{MSE} = \text{MSE}_{\text{early}} - \text{MSE}_{\text{late}} = \sigma_2^2 \left(1 - \frac{1}{(1 + \sigma_1^2/\sigma_\pi^2)(1 + \sigma_1^2/\sigma_\pi^2 + \sigma_2^2/\sigma_\pi^2)}\right) = \sigma_2^2 \left(1 - \frac{1}{1 + \Sigma A^2}\right)$$

(S10)

Where $\Sigma A^2$ is a sum of squares of various terms in the denominator. The presence of $\Sigma A^2$ in the denominator makes the ratio less than 1 and causes the whole expression to be positive. Based on this, we can conclude that $\text{MSE}_{\text{early}}$ is always larger than $\text{MSE}_{\text{late}}$ confirming that the late strategy leads to better performance.

**Length measurement and production task**

Subjects measured and produced visually presented lines drawn from an 11-point discrete uniform distribution between 10 and 15 degrees visual angle. To produce the length, subjects had to move a manipulandum underneath a horizontally positioned computer monitor. In each trial, after subjects positioned the manipulandum at the perimeter of the screen, a horizontal line flashed for 500 ms, after which subjects had

1200 ms to move the manipulandum inward to the final response position (**Supplementary Figure 4**). Two small vertical bars, one positioned at the initial location of the manipulandum and one tracking the horizontal location of the bar, provided online visual feedback during the response. The produced length was measured as the distance between the two vertical bars at the end of the response period. The gain in the identity and remapped contexts was 1 and 1.5, respectively. The gain was communicated by telling subjects at the start of the first session to produce either “the same as” or “one and a half times” the length of the sample. Response feedback was similar to the interval production task: following each response, the produced length was shown as a line between the marker bars (green for hit and red for miss), and the correct length was displayed immediately beneath in gray. Subjects completed four sessions total with each session comprising two blocks of 150 trials of identity and remapped trials for a total of 600 trials per session. The error threshold for each gain was on a one-up one-down staircase for the first two sessions, including the training session, and fixed for the final two sessions at the mean of the last 100 trials for each gain. The order of blocks associated with the identity and remapped blocks was counterbalanced across subjects.

In this task, the production stage might be subject to additional non-scalar execution noise due to hand movements, as previous work has suggested [2,3]. Therefore, we augmented the model we used in experiments 1 and 2 to include an additional signal independent noise term $\sigma_{post}$. We used this model to generate predictions for RMSE, BIAS, and √VAR for individual subjects in the remapped context under the assumption of no additional MTN. To do so, we simulated data using parameters of the augmented model ($w_{pre}$, $w_{post}$, and $\sigma_{post}$) fit in the identity context and calculated RMSE, BIAS, and √VAR from those data (**Supplementary Figure 4B,C**).

## Supplementary Tables

| | Identity context | | | | | | No additional SMN prediction | | | Remapped context | | | | | |
|---|---|---|---|---|---|---|---|---|---|---|---|---|---|---|---|
| Subject | RMSE | BIAS | √VAR | $w_{pre}$ | $w_{post}$ | offset | RMSE | BIAS | √VAR | RMSE | BIAS | √VAR | $w_{pre}$ | $w_{post}$ | offset |
| CK | 68 | 37 | 54 | 0.08 | 0.05 | 22 | 102 | 56 | 81 | 150 | 97 | 114 | 0.15 | 0.07 | 5 |
| JM | 99 | 71 | 67 | 0.15 | 0.06 | -20 | 148 | 106 | 100 | 156 | 86 | 115 | 0.12 | 0.08 | -38 |
| MW | 87 | 57 | 65 | 0.13 | 0.06 | 3 | 130 | 86 | 98 | 187 | 140 | 124 | 0.23 | 0.09 | -16 |
| RA | 90 | 48 | 76 | 0.11 | 0.08 | -3 | 136 | 71 | 115 | 187 | 101 | 111 | 0.15 | 0.08 | -80 |
| SMP | 81 | 28 | 73 | 0.07 | 0.08 | -14 | 121 | 42 | 110 | 195 | 113 | 145 | 0.16 | 0.11 | -41 |
| LZ | 76 | 45 | 61 | 0.10 | 0.06 | -4 | 114 | 67 | 91 | 178 | 111 | 137 | 0.17 | 0.10 | -17 |
| DS | 112 | 67 | 88 | 0.15 | 0.09 | -11 | 168 | 100 | 132 | 219 | 158 | 135 | 0.31 | 0.10 | -41 |
| BG | 87 | 53 | 67 | 0.11 | 0.07 | 18 | 130 | 79 | 101 | 189 | 143 | 111 | 0.24 | 0.09 | -35 |
| SC | 82 | 58 | 56 | 0.14 | 0.05 | -10 | 122 | 88 | 84 | 176 | 131 | 109 | 0.21 | 0.08 | -27 |
| SK | 74 | 39 | 64 | 0.08 | 0.06 | 15 | 112 | 59 | 96 | 132 | 90 | 97 | 0.12 | 0.06 | -1 |
| RP | 81 | 58 | 55 | 0.13 | 0.05 | -11 | 122 | 88 | 83 | 189 | 151 | 113 | 0.34 | 0.09 | -1 |

**Supplementary Table 1**. Subject performance and observer model parameters for the timing task in the identity and remapped contexts (gain = 1.5), along with performance predicted for the remapped context under the prediction of no additional SMN. All values are expressed in milliseconds, except for $w_{pre}$ and $w_{post}$, which are unitless.

| | Identity context | | | | | | No additional SMN prediction | | | Remapped context | | | | | |
|---|---|---|---|---|---|---|---|---|---|---|---|---|---|---|---|
| Subject | RMSE | BIAS | √VAR | $w_{pre}$ | $w_{post}$ | offset | RMSE | BIAS | √VAR | RMSE | BIAS | √VAR | $w_{pre}$ | $w_{post}$ | offset |
| CK | 68 | 37 | 54 | 0.08 | 0.05 | 22.12 | 51 | 28 | 40 | 78 | 58 | 50 | 0.17 | 0.05 | 21 |
| JM | 99 | 71 | 67 | 0.15 | 0.06 | -19.61 | 74 | 53 | 50 | 70 | 36 | 60 | 0.10 | 0.08 | 7 |
| MW | 87 | 57 | 65 | 0.13 | 0.06 | 2.75 | 65 | 43 | 49 | 90 | 51 | 75 | 0.15 | 0.10 | 28 |
| RA | 90 | 47 | 76 | 0.11 | 0.08 | -3.49 | 68 | 36 | 57 | 144 | 82 | 109 | 0.24 | 0.15 | 100 |
| SMP | 81 | 28 | 73 | 0.06 | 0.08 | -13.50 | 61 | 21 | 55 | 69 | 29 | 61 | 0.07 | 0.09 | -10 |
| LZ | 76 | 45 | 61 | 0.10 | 0.06 | -3.75 | 57 | 34 | 46 | 77 | 51 | 56 | 0.15 | 0.07 | -9 |
| DS | 112 | 67 | 88 | 0.15 | 0.09 | -10.89 | 84 | 50 | 66 | 80 | 53 | 59 | 0.18 | 0.08 | -3 |
| BG | 85 | 53 | 67 | 0.11 | 0.07 | 18.47 | 64 | 40 | 50 | 78 | 61 | 49 | 0.19 | 0.05 | -5 |
| AR | 102 | 56 | 82 | 0.13 | 0.08 | -12.78 | 77 | 42 | 62 | 115 | 78 | 81 | 0.30 | 0.13 | -19 |
| RG | 73 | 53 | 50 | 0.12 | 0.04 | -5.81 | 55 | 40 | 37 | 70 | 45 | 53 | 0.13 | 0.07 | -7 |
| SM3 | 121 | 92 | 79 | 0.23 | 0.08 | 3.73 | 91 | 69 | 59 | 124 | 79 | 90 | 0.23 | 0.12 | 87 |
| AM | 69 | 40 | 56 | 0.09 | 0.05 | -4.17 | 52 | 30 | 42 | 85 | 41 | 74 | 0.12 | 0.10 | -5 |

**Supplementary Table 2**. Subject performance and observer model parameters for the timing task in the identity and remapped contexts (gain = 0.75), along with performance predicted for the remapped context under the prediction of no additional SMN. All values are expressed in milliseconds, except for $w_{pre}$ and $w_{post}$, which are unitless.

| | Identity context | | | | | | Remapped context | | | | | |
|---|---|---|---|---|---|---|---|---|---|---|---|---|
| Subject | RMSE | BIAS | √VAR | $\sigma_{pre}$ | $\sigma_{post}$ | offset | RMSE | BIAS | √VAR | $\sigma_{pre}$ | $\sigma_{post}$ | offset |
| EG | 2.52 | 0.63 | 2.44 | 0.29 | 2.50 | 0.06 | 8.80 | 3.12 | 8.23 | 4.50 | 7.88 | 6.04 |
| JK | 2.86 | 0.72 | 2.76 | 0.31 | 2.82 | 0.30 | 8.62 | 6.05 | 6.15 | 11.15 | 4.31 | -0.39 |
| RC | 2.25 | 0.55 | 2.18 | 0.62 | 2.14 | -0.58 | 7.58 | 2.57 | 7.14 | 4.59 | 6.43 | 8.13 |
| DS | 2.30 | 0.52 | 2.24 | 0.68 | 2.18 | 0.20 | 11.64 | 6.26 | 9.81 | 11.13 | 8.35 | 13.47 |
| SMP | 1.73 | 0.36 | 1.70 | 0.35 | 1.68 | 0.05 | 7.01 | 4.03 | 5.74 | 6.57 | 4.55 | 3.81 |
| TT | 1.77 | 0.48 | 1.70 | 0.36 | 1.71 | 0.03 | 6.74 | 1.62 | 6.54 | 0.27 | 6.68 | 2.17 |
| JL | 2.51 | 0.55 | 2.44 | 0.23 | 2.49 | 0.80 | 7.40 | 3.54 | 6.50 | 6.09 | 5.33 | 3.55 |
| MW | 1.98 | 0.72 | 1.85 | 0.80 | 1.80 | -0.67 | 9.06 | 1.72 | 8.90 | 1.68 | 8.85 | 3.32 |
| SR | 3.26 | 0.37 | 3.24 | 0.27 | 3.23 | -0.53 | 9.55 | 5.30 | 7.95 | 9.24 | 6.72 | 2.27 |
| GH | 3.27 | 0.96 | 3.13 | 1.15 | 3.04 | 0.10 | 6.74 | 5.03 | 4.48 | 8.27 | 3.18 | 1.15 |
| CN | 2.12 | 0.38 | 2.09 | 0.27 | 2.09 | -0.28 | 7.93 | 3.81 | 6.96 | 6.38 | 5.86 | 2.64 |

**Supplementary Table 3**. Subject performance and observer model parameters for the center-out task in the identity and remapped contexts (60 deg visuomotor rotation). Performance predicted for the remapped context under the prediction of no additional SMN is equal to that in the identity context. All values are expressed in degrees.

| | Identity context | | | | | | No additional SMN prediction | | | Remapped context | | | | | |
|---|---|---|---|---|---|---|---|---|---|---|---|---|---|---|---|
| Subject | RMSE | BIAS | √VAR | $w_{pre}$ | $w_{post}$ | offset | RMSE | BIAS | √VAR | RMSE | BIAS | √VAR | $w_{pre}$ | $w_{post}$ | offset |
| SPA | 0.43 | 0.15 | 0.41 | 0.06 | 0.06 | -0.14 | 0.65 | 0.23 | 0.61 | 0.80 | 0.31 | 0.74 | 0.08 | 0.06 | 0.11 |
| OQB | 0.47 | 0.18 | 0.43 | 0.07 | 0.06 | 0.07 | 0.70 | 0.27 | 0.65 | 0.77 | 0.26 | 0.73 | 0.07 | 0.07 | -0.02 |
| AB | 0.40 | 0.14 | 0.37 | 0.04 | 0.06 | 0.09 | 0.60 | 0.21 | 0.56 | 0.82 | 0.48 | 0.67 | 0.10 | 0.06 | -0.06 |
| GQ | 0.41 | 0.13 | 0.39 | 0.00 | 0.08 | 0.15 | 0.62 | 0.20 | 0.59 | 0.76 | 0.47 | 0.60 | 0.10 | 0.05 | 0.01 |
| ]\[pMT | 0.38 | 0.12 | 0.36 | 0.03 | 0.06 | 0.10 | 0.56 | 0.18 | 0.54 | 0.68 | 0.36 | 0.57 | 0.07 | 0.05 | -0.02 |
| AK | 0.40 | 0.18 | 0.35 | 0.05 | 0.05 | 0.13 | 0.59 | 0.27 | 0.53 | 0.73 | 0.34 | 0.65 | 0.08 | 0.05 | 0.01 |
| BAL | 0.38 | 0.22 | 0.31 | 0.05 | 0.05 | 0.23 | 0.57 | 0.32 | 0.47 | 0.60 | 0.35 | 0.49 | 0.07 | 0.05 | -0.04 |
| SNH | 0.37 | 0.09 | 0.36 | 0.03 | 0.06 | 0.14 | 0.56 | 0.14 | 0.54 | 0.78 | 0.35 | 0.70 | 0.08 | 0.06 | 0.07 |
| DNA | 0.36 | 0.13 | 0.33 | 0.04 | 0.06 | 0.06 | 0.54 | 0.19 | 0.50 | 0.62 | 0.21 | 0.59 | 0.05 | 0.06 | 0.03 |
| EW | 0.37 | 0.20 | 0.32 | 0.06 | 0.05 | -0.06 | 0.56 | 0.30 | 0.47 | 0.75 | 0.45 | 0.60 | 0.09 | 0.06 | -0.08 |
| RC | 0.60 | 0.49 | 0.34 | 0.15 | 0.04 | 0.13 | 0.90 | 0.74 | 0.50 | 1.11 | 0.96 | 0.57 | 0.22 | 0.05 | -0.12 |
| AW | 0.54 | 0.17 | 0.52 | 0.00 | 0.10 | 0.42 | 0.81 | 0.25 | 0.77 | 0.95 | 0.43 | 0.85 | 0.09 | 0.07 | 0.31 |

**Supplementary Table 4**. Subject performance and observer model parameters for the length measurement and identification task in the identity and remapped contexts (gain = 1.5), along with performance predicted for the remapped context under the prediction of no additional SMN. All values are expressed in degrees visual angle, except for $w_{pre}$ and $w_{post}$, which are unitless.

## Supplementary references


1. Jazayeri M, Shadlen MN. Temporal context calibrates interval timing. Nat Neurosci. Nature Publishing Group; 2010;13: 1020–1026.

2. Wolpert DM, Ghahramani Z, Jordan MI. An internal model for sensorimotor integration. Science. 1995;269: 1880–1882.

3. van Beers RJ, Haggard P, Wolpert DM. The role of execution noise in movement variability. J Neurophysiol. 2004;91: 1050–1063.
